# Gut microbial diversity is associated with lower arterial stiffness in women

**DOI:** 10.1093/eurheartj/ehy226

**Published:** 2018-05-09

**Authors:** Cristina Menni, Chihung Lin, Marina Cecelja, Massimo Mangino, Maria Luisa Matey-Hernandez, Louise Keehn, Robert P Mohney, Claire J Steves, Tim D Spector, Chang-Fu Kuo, Phil Chowienczyk, Ana M Valdes

**Affiliations:** 1 Department of Twin Research and Genetic Epidemiology, King’s College London, St Thomas' Hospital, London, UK; 2 Division of Rheumatology, Allergy and Immunology, Chang Gung Memorial Hospital, Fuxing Street, Guishan Dist., Taoyuan City, Taiwan; 3 Department of Clinical Pharmacology, British Heart Foundation Centre, King’s College London, St Thomas' Hospital, London, UK; 4 NIHR Biomedical Research Centre at Guy’s and St Thomas’ Foundation Trust, St Thomas’ Hospital, London, UK; 5 Metabolon Inc., Research Triangle Park, NC, USA; 6 School of Medicine, Nottingham City Hospital, Hucknall Road, Nottingham, UK; 7 NIHR Nottingham Biomedical Research Centre, Queen's Medical Centre, Derby Rd, Nottingham, UK

**Keywords:** Gut microbiome diversity, Arterial stiffness, Microbial metabolites, Indolepropionate, Metabolic syndrome, Inflammation

## Abstract

**Aims:**

The gut microbiome influences metabolic syndrome (MetS) and inflammation and is therapeutically modifiable. Arterial stiffness is poorly correlated with most traditional risk factors. Our aim was to examine whether gut microbial composition is associated with arterial stiffness.

**Methods and results:**

We assessed the correlation between carotid-femoral pulse wave velocity (PWV), a measure of arterial stiffness, and gut microbiome composition in 617 middle-aged women from the TwinsUK cohort with concurrent serum metabolomics data. Pulse wave velocity was negatively correlated with gut microbiome alpha diversity (Shannon index, Beta(SE)= −0.25(0.07), *P* = 1 × 10^−4^) after adjustment for covariates. We identified seven operational taxonomic units associated with PWV after adjusting for covariates and multiple testing—two belonging to the *Ruminococcaceae* family. Associations between microbe abundances, microbe diversity, and PWV remained significant after adjustment for levels of gut-derived metabolites (indolepropionate, trimethylamine oxide, and phenylacetylglutamine). We linearly combined the PWV-associated gut microbiome-derived variables and found that microbiome factors explained 8.3% (95% confidence interval 4.3–12.4%) of the variance in PWV. A formal mediation analysis revealed that only a small proportion (5.51%) of the total effect of the gut microbiome on PWV was mediated by insulin resistance and visceral fat, c-reactive protein, and cardiovascular risk factors after adjusting for age, body mass index, and mean arterial pressure.

**Conclusions:**

Gut microbiome diversity is inversely associated with arterial stiffness in women. The effect of gut microbiome composition on PWV is only minimally mediated by MetS. This first human observation linking the gut microbiome to arterial stiffness suggests that targeting the microbiome may be a way to treat arterial ageing.

**Figure:**
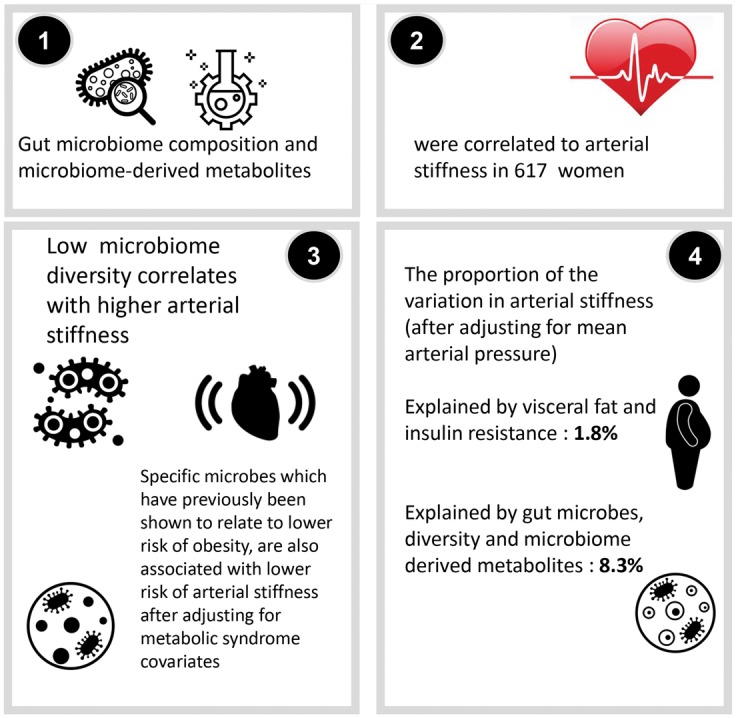

## Introduction

A substantial proportion of major adverse cardiovascular events (MACE) within the population are not explained by traditional cardiovascular risk factors. The gut microbiome has been implicated in a variety of potential disease mechanisms including oxidative stress and inflammation that could influence vascular disease.^1^^,^^2^ We therefore examined the relationship between the gut microbiome composition and arterial stiffness.

Arterial stiffness is an independent predictor of cardiovascular risk, especially in individuals with metabolic syndrome (MetS).^3^ Vascular stiffness is a consequence of pathophysiological alterations involving various functional elements of the vessel wall.^4^^,^^5^ It is a measure of vascular ageing predictive of MACE but (when adjusted for blood pressure) is weakly or unrelated to conventional risk factors.^6–8^

Several parameters are informative about arterial stiffness. Among these, pulse wave velocity (PWV) is currently considered the gold-standard measure of arterial stiffness and is predictive of future cardiovascular events.^9^ Both chronic hyperglycaemia and hyperinsulinaemia have been demonstrated to lead to hypertrophy of vascular smooth cells and fibrosis.^10^ In addition, levels of adipokines are significantly correlated with arterial stiffness.^11^ These associations suggest that factors contributing to insulin resistance and MetS may be involved in the development of arterial stiffness. Another important factor contributing to arterial stiffness is systemic inflammation.^12^ Evidence from this derives from the associations repeatedly reported between levels of c-reactive protein (CRP) and PWV in healthy individuals^13^^,^^14^ and from the higher levels of arterial stiffness seen in patients with primary inflammatory diseases after adjusting for cardiovascular risk factors.^15^^,^^16^

The gut microbiome, i.e. the community of microbes in the gastrointestinal tract,^17^^,^^18^ has recently emerged as an important regulator of systemic inflammation,^19^ glucose tolerance, and insulin sensitivity.^13^^,^^14^ We therefore hypothesized a relationship between gut microbiome composition and PWV. To test this, we first investigated the association between arterial stiffness, measured by PWV, and gut microbiome composition. We looked at the relationship between (i) arterial stiffness and loss of microbiome ‘diversity’ (loss in the number of species in the gut microbiome), a common finding in several disease states^1^^,^^20^ and (ii) between arterial stiffness and specific operational taxonomic units (OTUs). Secondly, we investigated whether any association between arterial stiffness and the microbiome might be accounted for by specific circulating metabolites known to be generated by the gut microbiome: phenylacetylglutamine and trimethylamine oxide (TMAO), previously linked to cardiovascular disease,^2^ and indoleproprionate (IPA),^21^ an antioxidant associated with MetS,^22^ and which might also influence arterial stiffness.

## Methods

Study subjects were female twins enrolled in the TwinsUK registry, a national register of adult twins recruited as volunteers without selecting for any particular disease or traits.^23^ Here, we analysed data from 617 female twins with PWV, serum metabolites, and gut microbiome composition determined by 16S rRNA gene sequencing.^24^ The study was approved by NRES Committee London–Westminster, and all twins provided written informed consent to take part in the study.

### Phenotype measurements

Brachial blood pressure was measured with the participants in a supine position according to British and Irish Hypertension Society Guidelines using a validated automated oscillometric device (Omron, 705 IT, Omron Health Care, Japan). Measurements were taken after at least 5 min of rest supine and an average of three measurements were used. Carotid-femoral PWV was calculated from sequential recordings of carotid and femoral artery pressure waveforms using the SphygmoCor system (AtCor medical, Australia). Difference in time of pulse arrival from the R-wave of the electrocardiogram between the two sites was taken as the transit time, and difference in path length was estimated using surface measurements as previously described.^25^ Measurements were made in triplicate, and mean values were used for analysis. The same system was used to obtain central and mean arterial blood pressure (MAP) by tonometric recording of radial artery pressure calibrated by brachial blood pressure. Estimates of visceral fat mass were derived from Dual-energy X-ray absorptiometry (DXA) measurements of whole body composition as previously described.^26^

Fasting insulin levels were measured for the twin cohort using the same methods as previously described.^26^ The homeostasis model assessment-estimated insulin resistance (HOMA-IR) was calculated multiplying overnight fasting plasma insulin (FPI) by overnight fasting plasma glucose (FPG), then dividing by the constant 22.5, i.e. HOMA-IR = (FPI × FPG)/22.515.

We calculated the 10-year atherosclerotic cardiovascular disease (ASCVD)^27^ to estimate the 10-year cardiovascular risk of an individual. The score is based on the individual age, sex, total and HDL cholesterol, systolic blood pressure, smoking status, use of blood pressure lowering medications, and the presence of type 2 diabetes^28^ and is calculated for women aged 40–79.

### Environmental risk factors

Dietary intakes were estimated from a validated 131-item food frequency questionnaire (FFQ).^29^ Fibre and omega 3 intakes (grams per day) were derived from the UK Nutrient Database,^30^ which provided food content of non-starch polysaccharides (NSP) determined by the Englyst method.^31^ Alcohol intake was measured by questionnaire and coded based on the average units consumption per week (0 = never, 1 =1-5 units per week, 2 = 6–10 units per week, 3 = 11–15 units per week, 4 = 16–20 units per week, 5 = 21–40 units per week, 6= >41+ units per week). Adherence to a Mediterranean diet was calculated using the modified Mediterranean diet score (MDS) method, as outlined by Trichopoulou *et al.*.^32^ Physical activity was measured by questionnaire asking their level of activity in a Likert scale (none, light, moderate, intense). In order to account for socioeconomic status, education, and access to health care an individual’s Index of Multiple Deprivation (IMD) score (2015) from the UK Office of National Statistics was used. This is based on the participants’ post code of residence (<seurld>https://www.gov.uk/government/statistics/english-indices-of-deprivation-2015</seurld>) (details in the Supplementary material online).

### Indoleproprionate, phenylacetylglutamine, and trimethylamine oxide measurement

Circulating serum levels of IPA, phenylacetylglutamine and TMAO were measured using ultra-high performance liquid chromatography-tandem mass spectrometry by the metabolomics provider Metabolon, Inc. (Research Triangle Park, USA) on fasting serum samples as described previously.^33^ We inverse normalized the metabolite data as it was not normally distributed. We imputed the missing values using the minimum run day measures.

### C-reactive protein measurement

*C-reactive protein* was measured by a highly sensitive automated microparticle capture enzyme immunoassay, standardized on the World Health Organization International Reference Standard for CRP immunoassay 85/506,^34^ as previously reported.^35^

### Microbiota analysis

A diagram summarizing the pipeline used in the analysis of stool samples for microbiome composition presented in Supplementary material online, *Figure S1*. A diagram of metabolomic profiling is presented in Supplementary material online, *Figure S2*.

The composition of the gut microbiome in faecal samples was determined by 16S rRNA gene sequencing carried out as previously described.^24^ Briefly, the V4 region of the 16S rRNA gene was amplified and sequenced on Illumina MiSeq. Reads were then summarized to operational taxonomic units (OTUs). Quality control was carried out on a per sample basis, discarding paired-ends with an overlap of less than 200 nt and removing chimeric sequences using de novo chimaera detection in USEARCH.^36^*De novo* OTU clustering was then carried across all reads using Sumaclust within Quantitative Insights Into Microbial Ecology (QIIME) 1.9.0, grouping reads with a 97% identity threshold.^37^^,^^38^ Quantitative Insights Into Microbial Ecology is a bioinformatic pipeline designated for the task of analysing microbial communities that were sampled through marker gene (e.g. 16S or 18S rRNA genes) amplicon sequencing. Sumaclust is a programme that clusters sequences and detects the ‘erroneous’ sequences created during amplification and sequencing protocols, deriving from ‘true’ sequences. OTU counts were converted to log transformed relative abundances, with zero counts handled by the addition of an arbitrary value (10^−6^). The residuals of the OTU abundances were taken from linear models, accounting for technical covariates including sequencing depth, sequencing run, sequencing technician, and sample collection method. These residuals were inverse normalized, as they were not normally distributed, and used in downstream analyses. In order to calculate alpha diversity, the complete OTU count table was rarefied to 10 000 sequences per sample 50 times. Alpha diversity metrics were calculated for each sample in each of the rarefied tables and final diversity measures taken as the mean score across all 50. Alpha diversities were quantified as observed OTU counts and Shannon and Simpson diversity indices (see the Supplementary material online for details). Alpha diversity indexes were standardized to have mean 0 and SD 1.

### Statistical analysis

Statistical analysis was carried out using Stata version 11. Random intercept logistic regressions were undertaken to evaluate the association between PWV and gut microbial diversity (Shannon and Simpson indexes and number of observed OTUs) adjusting for age, body mass index (BMI), mean arterial pressure (MAP, the component of blood pressure that is thought to influence PWV, rather than systolic blood pressure, which is in large part determined by PWV), and family relatedness.

Linear regression was also employed to investigate the association between PWV and OTUs adjusting for covariates, family relatedness, and multiple testing using false discovery rate (FDR < 0.1).

We accounted for familial relatedness using random intercept linear regression:

(1) $$Y_{i}=\beta_{0}+\beta_{i}X_{\mathrm{ij}}+\gamma_{i}{\text{age}}_{\mathrm{ij}}+\delta_{i}{\text{BMI}}_{\mathrm{ij}}+\rho_{j}\text{MAP}+\varsigma_{j}+\varepsilon_{\mathrm{ij}},$$

where *Y_i_* and *X_ij_* are respectively PWV (*Y*) and the microbiome abundance of twin *j* from pair *i*. *ζ*_*j*_ is the family-specific error component, which represents the omitted family characteristics or unobserved heterogeneity. The comparison between PWV and variables is performed between each twin pair. We reran the analyses additionally adjusting for (i) diet (omega 3 and fibre intake, adherence to a Mediterranean diet), (ii) environmental factors including smoking, alcohol drinking habits, physical activity, socioeconomic status, PPI, and antibiotics use, that have been associated with microbiota changes, (iii) insulin resistance (assessed by HOMA-IR), and visceral fat mass that are considered important factors affecting arterial stiffness and were associated with PWV in our data, (iv) the 10-year atherosclerotic cardiovascular disease (ASCVD) risk score^27^ to account for traditional CVD risk factors and serum levels of CRP to account for systemic inflammation, (v) uric acid, previously found to correlate with vascular stiffness.^39^ As faecal samples were taken 1.9 (SD = 1.5) years apart from the PWV measurement, we ensured that the associations between faecal microbiome composition and PWV were not influenced by the number of years between date of PWV measure and microbiome measure. We therefore (i) reran the analyses for the significant associations including samples taken more than 1 year apart and found no difference in the regression coefficients; (ii) further adjusted for the time elapsed between PWV measure and faecal sample collection and results were consistent.

We investigated for the association of three specific gut microbiome-derived metabolites: IPA, involved in insulin resistance, TMAO and phenylacetylglutamine and also adjusted for their levels.

Using standard multiple linear regressions, we computed the proportion of the variance (*R*^2^) and the corresponding 95% confidence intervals (95% CI)^40^ in PWV not explained by age, BMI, and MAP that was explained by microbiome diversity, microbiome OTUs, and microbiome-derived metabolites. We further employed partial least squares structural equation modelling (PLS-SEM)^41^ to test the mediation effects of ASCVD, HOMA + VF_mass_ and CRP (indirect effect) on the total effect of microbiome factors on PWV which was adjusted for age, BMI, and MAP (Supplementary material online, *Figure S3*). We constructed a mediation model to quantify both the direct effect of microbiome factors on PWV and the indirect (mediated) effects mentioned above. The model goodness of fit was assessed by standardized path coefficient and effect size (f^2^)^42–44^ yield the lower and upper bound of the 95% CI. The variance accounted for (VAF) score, which represents the ratio of indirect-to-total effect and determines the proportion of the variance explained by the mediation process, was further used to determine the significance of mediation effect.^41^ A separate model was built to assess the association between Shannon index and PWV. All PLS-SEM analysis was conducted using the Smart-PLS 3 software. We also used IBM SPSS AMOS software to conduct covariance-based (CB) SEM analysis. See Supplementary material online for more details.

## Results

**Figure 1:**
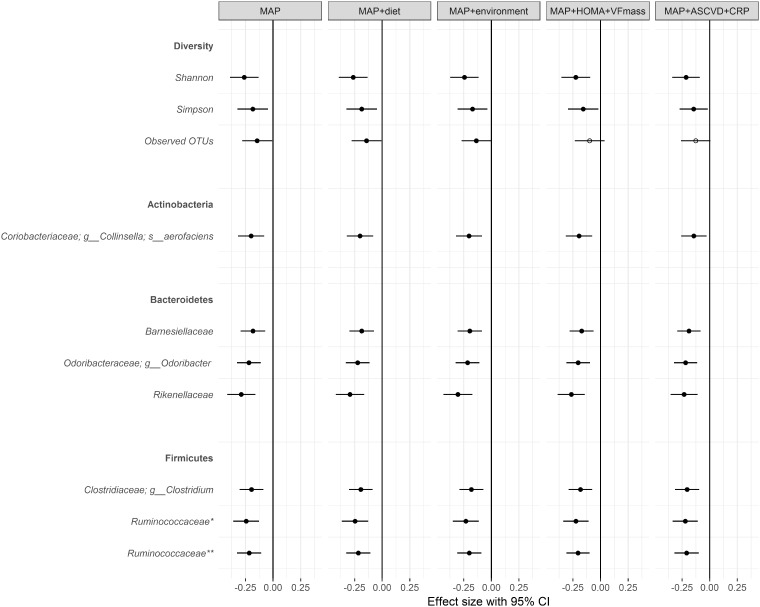
Microbes associated between pulse wave velocity and gut bacterial operational taxonomic units (false discovery rate < 0.1) adjusting for (i) age, body mass index, mean arterial blood pressure and family relatedness, (ii) age, body mass index, mean arterial blood pressure, fibre intake, omega 3 intake, adherence to a Mediterranean diet, and family relatedness, (iii) age, body mass index, mean arterial blood pressure, smoking, alcohol drinking habits, physical activity, PPI, antibiotics use, social deprivation status, and family relatedness, (iv) age, body mass index, mean arterial blood pressure, homeostasis model assessment-estimated insulin resistance, visceral fat mass, and family relatedness, (v) age, body mass index, mean arterial blood pressure, 10-years atherosclerotic cardiovascular disease risk score, c-reactive protein, and family relatedness.

The characteristics of the study participants are presented in *Table 1*. 617 females with both PWV and microbiome data were included in the analysis. Both Shannon and Simpson indices of gut microbiome diversity were significantly associated with PWV after adjusting for age, BMI, MAP, and family relatedness (*Figure 1*). Examining the association between PWV and bacterial lineages or OTUs identified 7 OTUs that were significantly and negatively associated with PWV after adjusting for covariates and multiple testing, FDR < 0.05 (*Figure 1* and Supplementary material online, *Table S1*). Two OTUs from the *Ruminococcaceae* family, one from the *Rikenellaceae* and one from the *Clostridiaceae*, a member of the Actinobateria *Collinsella aerofaciens*,^45^ a member of the *Barnesiellaceae* family, a member of the *Clostridiaceae* family, and the genus *Odoribacter* were negatively correlated with PWV.

**Table 1:** Descriptive characteristics of the study population

Phenotype		
	*N*	%
*N*	617	
Females (%)	617	100
Physical activity		
Low, *n* (%)	89	14.47
Medium or high, *n* (%)	528	85.53
T2D		
Yes, *n* (%)	25	4
No, *n* (%)	592	96
Use of antibiotics		
Yes, *n* (%)	10	1.57
No, *n* (%)	207	98.43
Use of PPIs		
Yes, *n* (%)	91	14.78
No, *n* (%)	526	85.22
	Mean	SD
Age (years)	61.42	7.34
10-years ASCVD risk score	7.37	6.63
BMI (kg/m^2^)	26.33	4.67
CRP (mmol/L)	2.71	6.45
DBP (mmHg)	78.02	8.72
Fibre intake (g/day)	20.85	6.09
HOMA-IR	0.96	0.68
IMD	6.96	2.38
MAP (mmHg)	94.62	10.02
MDS	4.58	1.70
Omega 3 intake (g)	1.62	0.62
PWV (m/s)	9.39	1.86
SBP (mmHg)	127.79	15.77
VFAT mass (g)	585.79	275.66
Indices of microbiome diversity		
Number of observed OTUs	309.27	87.83
Shannon diversity	5.19	0.71
Simpson diversity	0.93	0.066

DBP, diastolic blood pressure; PPI, proton pump inhibitors; SBP, systolic blood pressure; T2D, type 2 diabetes; VFAT, visceral fat.

Smoking/alcohol drinking habits, physical activity, fibre and omega 3 intake, adherence to a Mediterranean diet, socioeconomic status, PPI, and antibiotics use, have been associated with either microbiota changes^46–49^ or with arterial stiffness.^50–53^ Therefore, we additionally adjusted for these risk factors as potential confounders and found that the results remain consistent. Because levels of uric acid have been associated with arterial stiffness^39^ we also adjusted for this factor (*Figure 1* and Supplementary material online, *Table S1*). In addition to lifestyle factors, we also adjusted for traditional cardiovascular risk factors and for systemic inflammation (*Figure 1* and Supplementary material online, *Table S1*). To do this, we used the 10-year ASCVD risk score computed for each individual and CRP levels. The results remain unchanged by these adjustments (*Figure 1*).

Because both higher gut microbiome diversity and the relative abundance of microbes are associated with lower visceral fat mass^54^^,^^55^ and insulin resistance,^56^ we assessed whether these associations were simply caused by higher visceral fat or higher insulin resistance. Pulse wave velocity remained significantly associated with both microbiome diversity and the identified microbial lineages (*Figure 1*) after adjusting for these measures. Thus, in our cohort, PWV is significantly associated with gut microbiome composition after adjusting for the known likely covariates (insulin resistance, visceral fat) with each standard deviation of the microbial abundances contributing an effect size of -0.12 to -0.27 on PWV and achieving p-values ranging from 0.002 to 3 × 10^−5^ (Supplementary material online, *Table S1*).

We also explored whether specific compounds known to be generated by the gut microbiome that could be implicated in arterial ageing, like phenylacetylglutamine,^57^ TMAO,^2^ and the antioxidant IPA^21^ were related to arterial stiffness. The associations between PWV and gut microbiome diversity and specific OTUs remained significant after adjustment for these three compounds, although some of the associations were slightly attenuated suggesting that in part some of the effects of the gut microbiome on arterial stiffness are mediated by levels of phenylacetylglutamine and IPA.

Next, we quantified how much of arterial stiffness could be explained by gut microbiome composition, and two of the three compounds generated by the gut microbiome linked to this cardiovascular trait. We linearly combined the PWV-associated gut microbiome-derived variables—diversity measures (Shannon, Simpson, and number of OTUs), bacterial OTUs, circulating levels of the microbiome-derived metabolites phenylacetylglutamine, and IPA. After adjusting for age, BMI, and mean arterial pressure the overall proportion of variance explained by microbiome factors is 8.3% (95% CI 4.32–12.4%).

**Figure 2:**
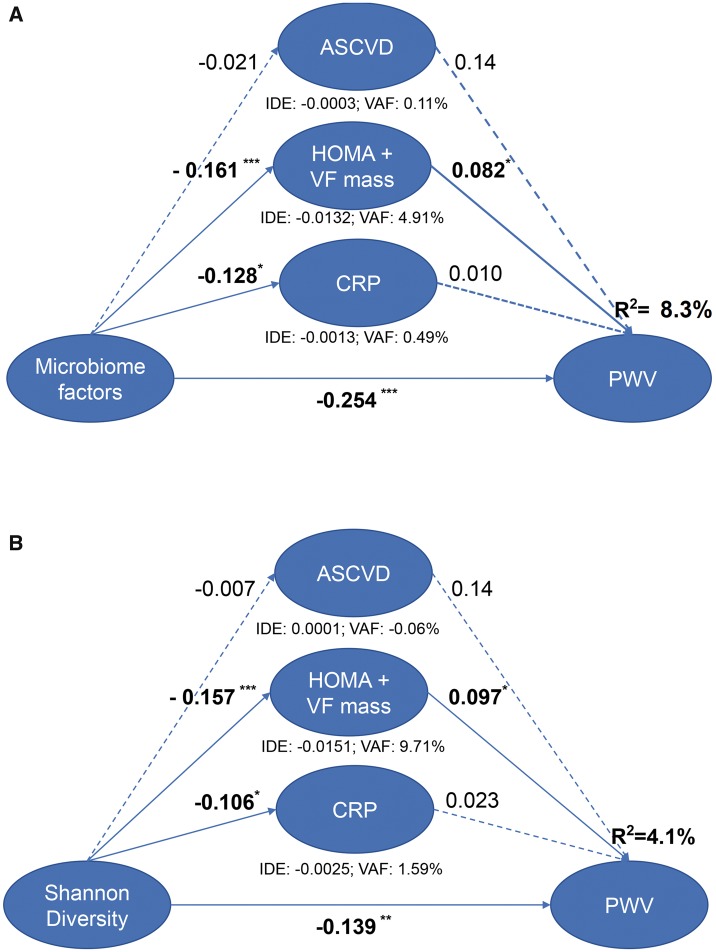
Mediation analysis of the association between (*A*) microbiome factors and (*B*) Shannon diversity and pulse wave velocity using partial least squares structural equation modelling. Path coefficients are denoted beside each path and indirect effect and variance accounted for (variance accounted for) score is denoted below each mediator (*P < 0.05; **P < 0.01; ***P < 0.001).

We finally conducted PLS-SEM analysis to determine the indirect effect of ASCVD, HOMA + VFmass, and CRP on the effect between microbiome factors and PWV. The mediation model (*Figure 2A*), which intends to evaluate the strength of the indirect effects, found that the direct relationship between microbiome factors and PWV was statistically significant (path coefficient = −0.254, *P* < 0.001) and the overall *R*^2^ was 8.4%. For indirect effect, only the effect of HOMA + VFmass was statistically significant. The VAF score for HOMA + VFmass was 4.91% while the combined VAF score of ASCVD, HOMA + VFmass, and CRP was 5.51%. The model to assess the relationship between Shannon diversity and PWV also found a significant direct effect (*R*^2^ 4.1%; path coefficient −0.139; *P* < 0.001). The composite indirect effect was 11.24% (*Figure 2B*). We also conducted CB-SEM analysis and found similar results (see Supplementary material online, *Figure S4*). Thus, both PLS-SEM and CB-SEM analyses found that the indirect effect of the gut microbiome mediated by CRP, HOMA + VFmass, and ASCVD risk to be statistically significant but of a small magnitude, with the majority of the effect of gut microbiome composition on PWV not being mediated by these factors.

**Take home figure:**
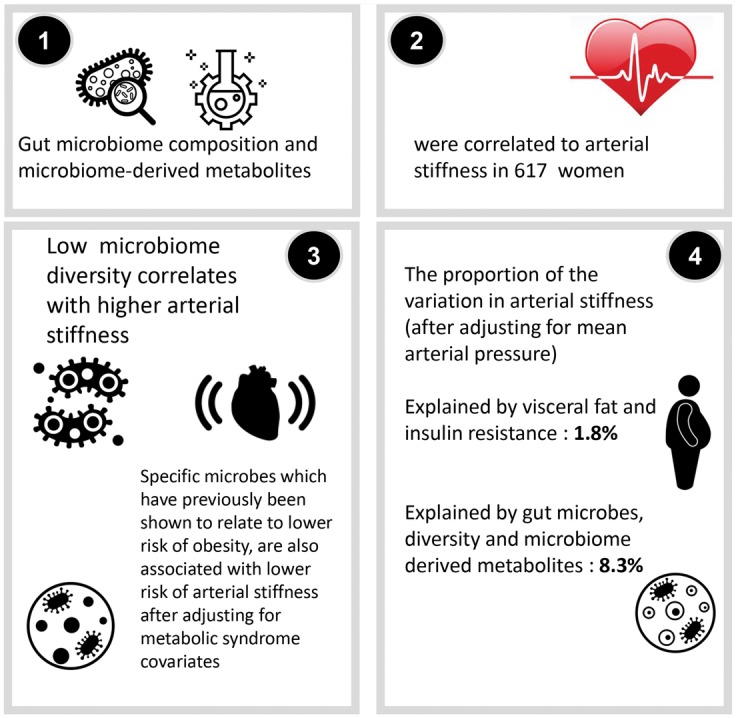
The gut microbiome is related to metabolic syndrome and inflammation, is modifiable via diet, medication and probiotics. Arterial stiffness (measured by pulse wave velocity) is a predictor of major cardiovascular events, which is related to metabolic syndrome and inflammation but poorly correlated with most traditional risk factors other than mean arterial pressure. The hypothesis of this study was that the gut microbiome composition could be related to arterial stiffness. This was measured in 617 women and both specific microbes and gut microbiome diversity, a measure of gut dysbiosis, along with metabolites generated by the gut microbiome were found to be associated with arterial stiffness. In fact, the microbiome related factors explain 8.3% of the variance in pulse wave velocity compared with only 1.8% of insulin resistance combined with visceral fat. These data indicate a strong contribution of the gut microbiome to risk of arterial stiffness and suggest targeting the gut microbiome composition as a therapeutic strategy.

## Discussion

The major novel finding of this study is that arterial stiffness as measured by carotid-femoral PWV is inversely correlated with gut microbiome diversity and with the abundance of specific microbes in the gut. These associations appear to be only in a small proportion mediated by an effect on MetS-related traits such as insulin resistance or visceral fat, but appear to be stronger than the associations of MetS and CRP in with PWV.

Specifically, we find that arterial stiffness correlates negatively with the abundance of *Ruminococcaceae* family bacteria. These are butyrate-producing bacteria whose abundance has been shown in mice to be linked to lower endotoxemia.^58^ Experimentally induced acute endotoxaemia is known to increase inflammatory cytokines and to cause endothelial dysfunction in humans, and chronic endotoxaemia is associated with MetS.^59^ It is well known that obesity, higher visceral fat, and insulin resistance all correlate with lower microbiome diversity.^56^ These factors contribute to arterial stiffness, but microbiome diversity remains negatively correlated with PWV after adjustment for all these factors. Similarly, microbiome-derived metabolites, such as phenylacetyl glutamine, are associated with lower PWV, but the association between OTUs and diversity with PWV remains significant even after adjusting for these microbiome-derive metabolites. We also find that all the microbiome factors that we identified, namely diversity, seven OTUs, and two microbiome-derived metabolites, make a contribution to explaining variation in PWV (*Figure 2*) and only a small proportion of that effect can be explained (after adjusting for MAP, age, and BMI) by traditional risk factors, MetS and CRP levels. However, it is known that inflammatory markers are very strongly associated with arterial stiffness^60^ and, although we find that only a small proportion of the effect of microbiome composition on PWV, CRP is not the only known biomarker for systemic inflammation predictive of CVD. Other markers, such as interleukin (IL)-6^61^ are associated with increased risk in addition to the inflammation measured by CRP. Moreover, a meta-analysis of up to 29 population-based prospective studies, IL-6, IL-18, and TNF-α were all found to result in significantly higher relative risks for non-fatal myocardial infarction or CHD death after adjusting for traditional risk factors.^62^

The causal role of inflammation in the pathogenesis of cardiovascular disease^63^ has been clearly demonstrated recently by the CANTOS trial (Canakinumab Anti-Inflammatory Thrombosis Outcomes Study).^64^ This trial has demonstrated beyond doubt that inflammation plays a role in the development of atherothrombosis by providing robust evidence that inhibiting IL-1β reduces the incidence of repetitive atherothrombotic events.

The fact that the gut microbiome composition plays a key role in inflammatory and autoimmune disease is now well characterized.^65^ A common theme in inflammatory diseases, ranging from rheumatoid arthritis, psoriasis, inflammatory bowel disease, and multiple sclerosis, is a reduced microbiome diversity.^65^ Although, clearly, functional characterization of the role of the various microbial lineages and microbial-derived compounds on arterial stiffness is needed, we hypothesize that the effect of gut microbiome composition on PWV is likely to be due caused by its role in modulating systemic inflammation and that only a part of this effect is captured by measuring CRP, insulin resistance, and traditional risk factors part of the 10-years ASCVD risk score.

This is relevant because the gut microbiome’s diversity and composition is modifiable. Gut microbial richness and composition are, to a large extent, modulated by diet.^66^ There is increasing evidence that insufficient consumption of dietary fibre leads to a loss of bacterial species in the human gut.^67^ Probiotics (live micro-organisms that influence the gut microbiome, mostly *Bifidobacterium* and *Lactobacillus* species) are another method to target the gut microbiome. A recent systematic review has shown that probiotic supplementation has a beneficial effect on blood pressure.^68^

We note several strengths and limitations to the current study. The study was based on middle-aged white female twins and hence may not be generalizable to other ethnic groups or to men. Although the characteristics of these women are representative of the general UK female population,^23^ clearly studies in men and in other ethnic groups are needed.

The faecal samples collected were not necessarily taken at the time of the PWV assessment. However, after adjustment for the time elapsed between the two measures we find no difference in the observed associations. For this reason, we suggest that our data are most likely valid, which would be consistent with data showing that the taxonomic composition and diversity of the gut microbiome remain constant over time^69^ in the absence of gross perturbation.^70^ It is postulated in the literature that long-term stability of the human indigenous microbial communities is maintained not by inertia but by the action of restorative forces within a dynamic system.^70^ Hence, the reported associations between gut microbiome composition and arterial stiffness unlikely to change majorly over time in a given individual.

Another limitation is the cross-sectional nature of the data. While there is biological plausibility via endotoxaemia for the association between arterial stiffness and microbiome composition being causal, this cannot be concluded from a cross-sectional study. On the other hand, we note several strengths, including the sample size of the study and the detailed clinical and molecular phenotyping of the study subjects, which has allowed us to test the relative contributions of different factors to arterial stiffness.

Here, we show that cardiovascular risk that is not explained by classical risk factors is likely to be in part captured by characterization of the microbiome and may in the future help stratify CVD risk, particularly in younger individuals and in women. Although it is unlikely to contribute to cardiovascular prevention guidelines at present, the findings presented here fit within the context of current European guidelines^71^ as follows: (i) The key outcome analysed was PWV. The Sixth Joint Task Force of the European Society of Cardiology concluded that PWV may serve as a useful biomarker to improve CVD risk prediction for patients close to decisional thresholds, although its systematic use in the general population to improve risk assessment is not recommended. (ii) One of the gaps in evidence identified by the European Task Force is that women continue to be under-represented in clinical trials. This study focuses specifically on cardiovascular risk in women. (iii) Gut microbiome composition is a modifiable factor influenced by dietary fibre intake. Fibre intake is part of the current recommendations for a healthy diet in the 2016 Task Force recommendation. In fact, the gut microbiome composition may contribute to the mechanism whereby dietary fibre intake influences cardiovascular risk, which is yet to be fully elucidated.

In conclusion, in this study we show for the first time that the composition of the gut microbiome is strongly correlated with levels of arterial stiffness in women independently of visceral fat and other obesity-related traits. Given the possibility of modifying the gut microbiome composition via diet and probiotic supplementation, this opens therapeutic avenues for reducing arterial stiffness targeting the gut microbiome.

## Supplementary material

Supplementary material is available at *European Heart Journal* online.

## Supplementary Material

Supplementary Data
