# Supplementary material for: Gut microbial diversity is associated with lower arterial stiffness in women
Source: Eur Heart J. 2018 May 9;39(25):2390–7. doi: 10.1093/eurheartj/ehy226 (PMC6030944; doi:10.1093/eurheartj/ehy226)
Supplement: Supplementary Data [file ehy226_suppl_data.docx]

**Gut microbial diversity is associated with lower arterial stiffness in women**

Cristina Menni^1^, Chihung Lin^2^, Marina Cecelja^3^, Massimo Mangino^1^, Maria Luisa Matey-Hernandez^1^, Louise Keehn^3^, Robert P Mohney^4^, Claire J Steves^1^, Tim D Spector^1^, Chang-Fu Kuo^2, 5^, Phil Chowienczyk^3^, Ana M Valdes ^1 5,6,*^

**Supplementary Material**

**Supplementary Table 1.** Association between PWV and gut bacterial operational taxonomic units (FDR<0.05) adjusting for (i) age, BMI, MAP and family relatedness, (ii) age, BMI, MAP, fibre intake, omega 3 intake, adherence to a Mediterranean diet and family relatedness,(iii) age, BMI, MAP, smoking, alcohol drinking habits, physical activity, PPI, antibiotics use, socioeconomic status (index of multiple deprivation) and family relatedness, (iv) age, BMI, MAP, visceral fat, HOMA-IR and family relatedness, (v) age, BMI, MAP, 10-years ASCVD risk and family relatedness, (vi) age, BMI, MAP, urate and family relatedness, (vii) age, BMI, MAP, TMAO, IPA, phenyl (phenylacetylglutamine) and family relatedness.

|  |  | MAP | | +diet | | +environ | | +HOMA+VFmass | | +ASCVD+CRP | | +urate | | +IPA+Pheny  +TMAO | |
| --- | --- | --- | --- | --- | --- | --- | --- | --- | --- | --- | --- | --- | --- | --- | --- |
|  | **Taxa** | **Beta**  **(SE)** | **P** | **Beta**  **(SE)** | **P** | **Beta**  **(SE)** | **P** | **Beta**  **(SE)** | **P** | **Beta**  **(SE)** | **P** | **Beta**  **(SE)** | **P** | **Beta**  **(SE)** | **P** |
|  | ***Diversity*** |  |  |  |  |  |  |  |  |  |  |  |  |  |  |
|  | Shannon | -0.26  (0.07) | 9.05x10^-5^ | -0.26  (0.07) | 8.24x10^-5^ | -0.24  (0.07) | 2.22x10^-4^ | -0.22  (0.07) | 8.05x10^-4^ | -0.21  (0.06) | 9.32x10^-4^ | -0.25  (0.07) | 1.41x10^-4^ | -0.24  (0.07) | 4.96x10^-4^ |
|  | Simpson | -0.18  (0.07) | 9.93x10^-3^ | -0.19  (0.07) | 8.72x10^-3^ | -0.17  (0.07) | 1.38x10^-2^ | -0.16  (0.07) | 2.48x10^-2^ | -0.15  (0.07) | 2.68x10^-2^ | -0.18  (0.07) | 1.32x10^-2^ | -0.16  (0.07) | 1.66x10^-2^ |
|  | Observed OTU | -0.14  (0.07) | 3.75x10^-2^ | -0.14  (0.07) | 4.11x10^-2^ | -0.14  (0.07) | 4.66x10^-2^ | -0.1  (0.07) | 1.53x10^-1^ | -0.13  (0.07) | 6.45x10^-2^ | -0.14  (0.07) | 4.83x10^-2^ | -0.12  (0.07) | 8.66x10^-2^ |
|  |  |  |  |  |  |  |  |  |  |  |  |  |  |  |  |
| *Internal ID* | ***OTU*** |  |  |  |  |  |  |  |  |  |  |  |  |  |  |
| *denovo44* | k__Bacteria; p__Bacteroidetes; c__Bacteroidia; o__Bacteroidales; f__Rikenellaceae; g__; s__ | -0.29  (0.07) | 1.41x10^-5^ | -0.29  (0.07) | 1.09x10^-5^ | -0.3  (0.07) | 7.27x10^-6^ | -0.27  (0.06) | 3.35x10^-5^ | -0.23  (0.06) | 2.19x10^-4^ | -0.28  (0.07) | 2.54x10^-5^ | -0.28  (0.06) | 2.39x10^-5^ |
| *denovo47* | k__Bacteria; p__Firmicutes; c__Clostridia; o__Clostridiales; f__Ruminococcaceae; g__; s__ | -0.24  (0.06) | 5.74x10^-5^ | -0.25  (0.06) | 6.27x10^-5^ | -0.23  (0.06) | 1.51x10^-4^ | -0.22  (0.06) | 1.63x10^-4^ | -0.22  (0.06) | 1.70x10^-4^ | -0.23  (0.06) | 9.82x10^-5^ | -0.22  (0.06) | 2.64x10^-4^ |
| *denovo381* | k__Bacteria; p__Firmicutes; c__Clostridia; o__Clostridiales; f__Ruminococcaceae; g__; s__ | -0.22  (0.06) | 1.37x10^-4^ | -0.22  (0.06) | 1.27x10^-4^ | -0.2  (0.06) | 3.80x10^-4^ | -0.2  (0.05) | 1.63x10^-4^ | -0.21  (0.06) | 2.10x10^-4^ | -0.2  (0.06) | 2.85x10^-4^ | -0.2  (0.06) | 6.80x10^-4^ |
| *denovo82* | k__Bacteria; p__Bacteroidetes; c__Bacteroidia; o__Bacteroidales; f__[Odoribacteraceae]; g__Odoribacter; s__ | -0.22  (0.05) | 8.14x10-5 | -0.22  (0.05) | 6.15x10^-5^ | -0.22  (0.05) | 1.01x10^-4^ | -0.2  (0.05) | 2.51x10^-4^ | -0.22  (0.05) | 5.06x10^-5^ | -0.21  (0.05) | 1.27x10^-4^ | -0.22  (0.05) | 4.25x10^-5^ |
| *denovo52* | k__Bacteria; p__Firmicutes; c__Clostridia; o__Clostridiales; f__Clostridiaceae; g__Clostridium; s__ | -0.2  (0.05) | 4.33x10^-4^ | -0.19  (0.05) | 4.42x10^-4^ | -0.18  (0.06) | 1.16x10^-3^ | -0.18  (0.05) | 9.04x10^-4^ | -0.2  (0.06) | 2.86x10^-4^ | -0.19  (0.05) | 6.23x10^-4^ | -0.18  (0.06) | 1.67x10^-3^ |
| *denovo94* | k__Bacteria; p__Actinobacteria; c__Coriobacteriia; o__Coriobacteriales; f__Coriobacteriaceae; g__Collinsella; s__aerofaciens | -0.2  (0.06) | 1.21x10^-3^ | -0.2  (0.06) | 1.03x10^-3^ | -0.2  (0.06) | 8.21x10^-4^ | -0.2  (0.06) | 1.43x10^-3^ | -0.14  (0.06) | 1.46x10^-2^ | -0.2  (0.06) | 1.07x10^-3^ | -0.19  (0.06) | 2.09x10-3 |
| *denovo50* | k__Bacteria; p__Bacteroidetes; c__Bacteroidia; o__Bacteroidales; f__[Barnesiellaceae]; g__; s__ | -0.18  (0.06) | 1.45x10^-3^ | -0.19  (0.06) | 1.28x10^-3^ | -0.2  (0.06) | 6.23x10^-4^ | -0.17  (0.06) | 2.18x10^-3^ | -0.19  (0.05) | 5.64x10^-4^ | -0.18  (0.06) | 1.88x10^-3^ | -0.17  (0.06) | 2.21x10^-3^ |

**Supplementary material**

**Socioeconomic status and index of multiple deprivation:**

Each individual was assigned an Index of Multiple Deprivation (IMD) score (2015) for each LSOA (from the Office of National Statistics) based on their post code of residence. The English Indices of Deprivation 2015 are based on 37 separate indicators, organised across seven distinct domains of deprivation (Income Deprivation; Employment Deprivation; Health Deprivation and Disability; Education, Skills and Training Deprivation; Crime; Barriers to Housing and Services; and Living Environment Deprivation) which are combined, using appropriate weights, to calculate the Index of Multiple Deprivation 2015 (IMD 2015). This is an overall measure of multiple deprivation experienced by people living in an area and is calculated for every neighbourhood, in England. Every such neighbourhood in England is ranked according to its level of deprivation relative to that of other areas( https://www.gov.uk/government/statistics/english-indices-of-deprivation-2015).

**Calculation of alpha diversity:**

Alpha diversity is an ecological measure of the mean species diversity of the community within one site (or one sample), i.e., the number of species and their proportion within one sampling site. Some commonly used indices to describe alpha diversity include Shannon index (H), Simpson's index (D) and the total number of species^1^. If we denote R the total number of OTUs (operational taxonomic units) the Shannon index is given by

$$H^{'}=\sum_{i=1}^{R} p_{i}lnp_{i}$$

Where p_i_ is the relative abundance of the ith OTU. The Simpson index is given by

$$1-D=\sum_{i=1}^{R} {p_{i}^{2}}$$

**Structural equation modelling and mediation analysis**

In this study, we used structural equation modelling (SEM) to observe the effect of mediators. We assessed the effect of microbiome factors or Shannon diversity on PWV and further estimate the direct and indirect effects mediated by ASCVD, HOMA-IR + VFmass and CRP. SEM is a multivariate statistical analysis method to analyse structural relationship (multiple paths) between measured and latent variables. It is particularly useful to analyse the effect of mediators and moderators. SEM can be broadly classified in partial least square (PLS)-SEM and covariance-based (CB) SEM. While PLS-SEM is generally used to do exploratory and CB-SEM confirmatory analysis, the distinction is not very clear. In this study, we conducted both PLS-SEM and CB-SEM.

The basic model is shown in **Supplementary Figure 3**. The calculation of indirect effect is the product of two direct effects (a and b) and c is direct effect between independent and outcome variables. Hair et al.^2^ advise to use variance accounted for (VAF) score to determine the magnitude of indirect effect. The formula of VAF calculation:

$$VAF=\frac{a*b}{a*b+c}$$

VAF estimates the proportion of indirect effect to total effects. In general, a VAF score less than 20% suggests a low indirect effect^3^.

The main SEM analysis was based on PLS-SEM using smartPLS 3 software. We conducted PLS-SEM as an exploratory analysis since PLS-SEM is more robust for data that are not normally distributed. In general, PLS SEM is more suitable for exploratory analysis and CB-SEM is more suitable for confirmatory study. In this study we use effect size (f^2^ = 0.019, > 0 generally have good effect size), variance inflation factor (for all covariates < 5) and predictive relevance (Q^2^ , 0.003-0.022 in the model all >0, indicates good quality) to assess model fit and all indices indicates good model fit^4, 5^. We also conducted CB-SEM based on AMOS 18 software. The results are similar to the main analyses and are shown in the following paragraph.

**CB SEM analysis using AMOS**

We also conducted CB-SEM analysis to confirm the results of PLS-SEM regarding the indirect effect of ASCVD, HOMA-IR +VFmass and CRP on the effect between microbiome factors and PWV. The CB-SEM analysis used AMOS 18 software. We used bootstrapping analysis with 5000 iterations which is similar to the main PLS-SEM analysis. The path coefficients for the microbiome factors and Shannon diversity models are close to the estimates made by PLS-SEM (S**upplementary Figure 4a and 4b**). The mediation model found that the direct relationship between microbiome factors and PWV was also statistically significant (path coefficient = -0.834, p < 0.001) and the overall R^2^ was 8.2%. The overall indirect effect was 4.91% of the total effect. The model to assess the relationship between Shannon diversity and PWV also found a significant direct effect (R^2^, 3.8%; path coefficient, -0.201; p<0.001). The composite indirect effect was 11.38% (figure 2b). According to Hair et al, the indirect effect in both models are low^2^, which is consistent to the PLS-SEM analysis^3^.

**Supplementary Figures**

**Supplementary Figure 1. Graphic representation of the study analysis pipeline followed. Participants from the TwinsUK cohort donated a faecal sample from which DNA was extracted. The 16S rRNA gene was amplified, aligned and matched to existing databases and microbial lineages (“operational taxonomic units” or OTUs) were assigned. Given the relative abundances of these OTUs we calculated “alpha diversity” measures, such as the total number of OTUs or the Shannon and Simpson’s indices, which reflect how many different OTUs are present in each individual and how abundant they are. The relative abundances of the OTUs were also transformed into inverse normal variables to be able to assess the correlation between these parameters of gut microbiome composition and arterial stiffness.**


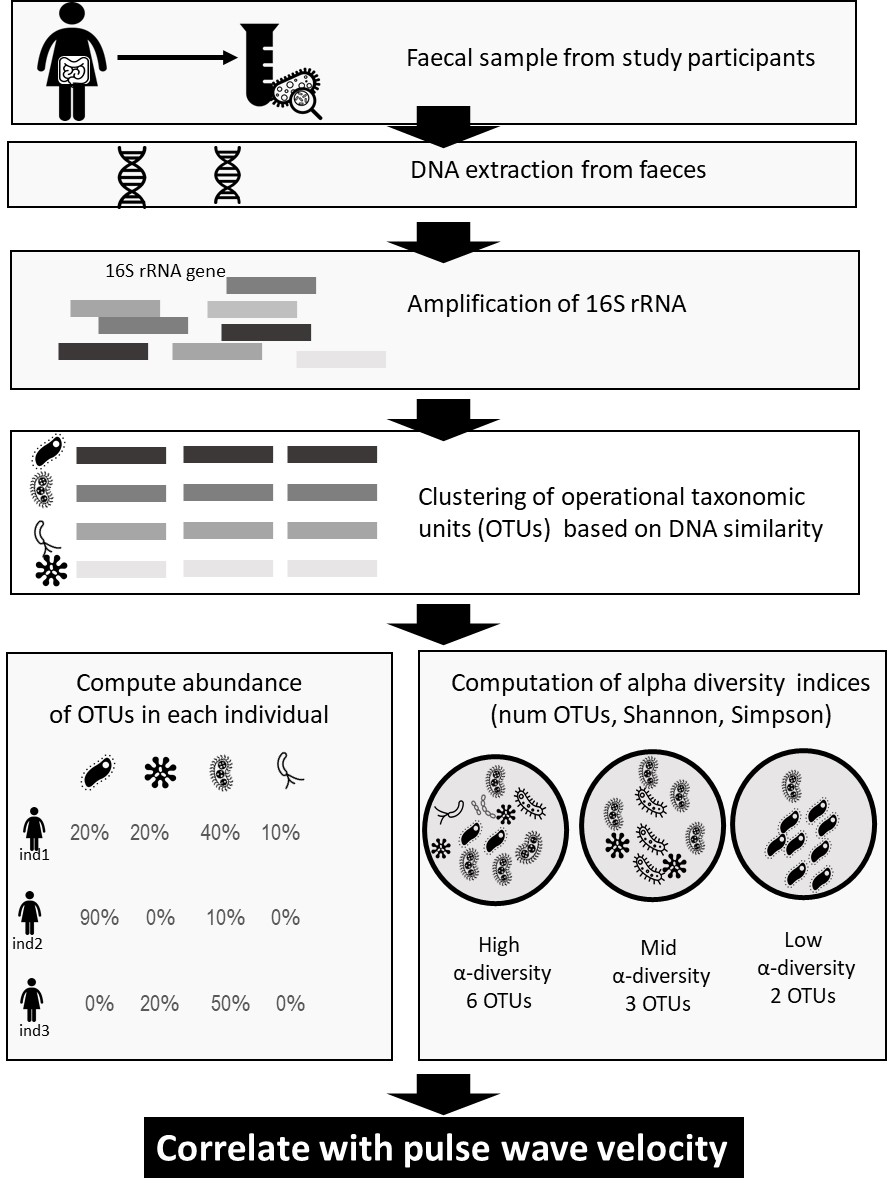


**Supplementary Figure 2. Graphic representation of metabolomic profiling**

**
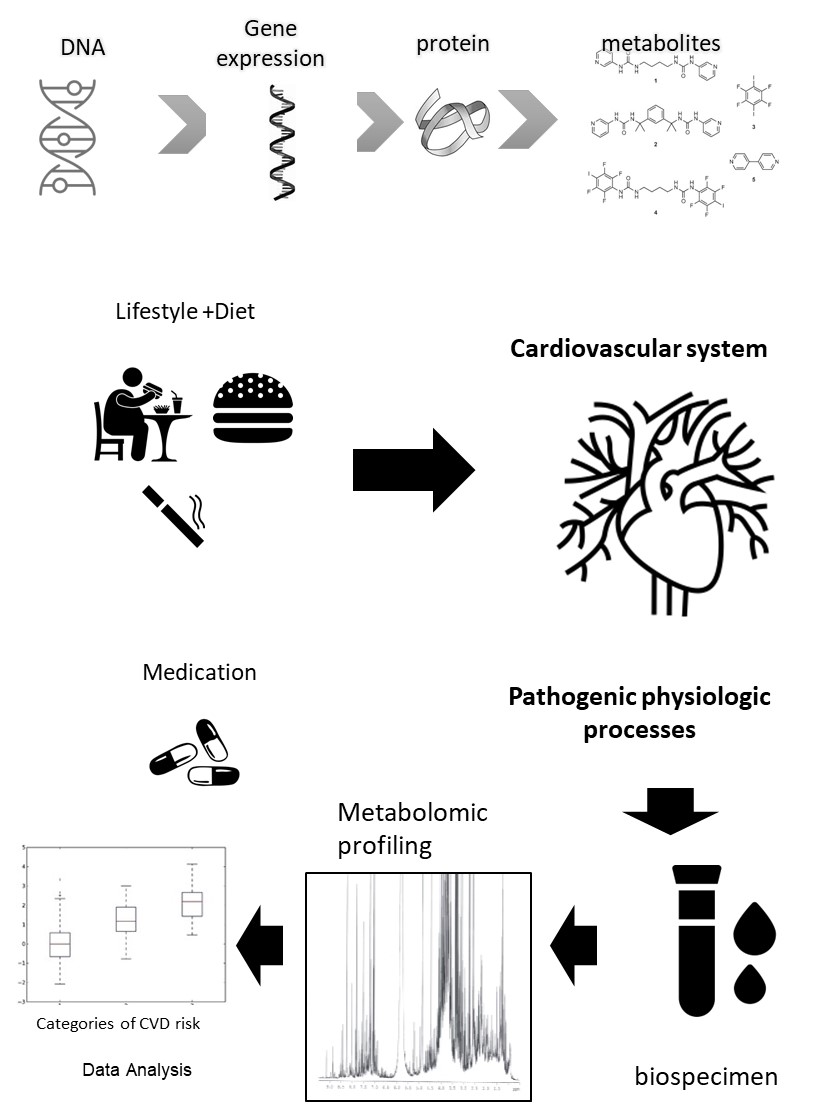
**

**Supplementary Figure 3: Basic model for mediator effect in SEM.**

a

Independent Variable

Outcome Variable

b

Mediator

c

Figure X: Mediator model

**Supplementary Figure 4:** Mediation analysis of the association between (a) microbiome factors and (b) Shannon diversity and PWV using CB-SEM. Path coefficients are denoted beside each path (*, p<0.05; **, p<0.01; ***, p<0.001).

(a)


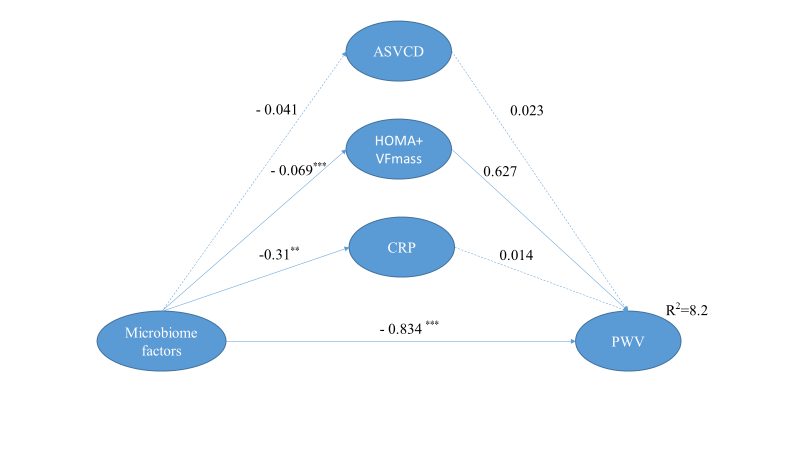


**(b)
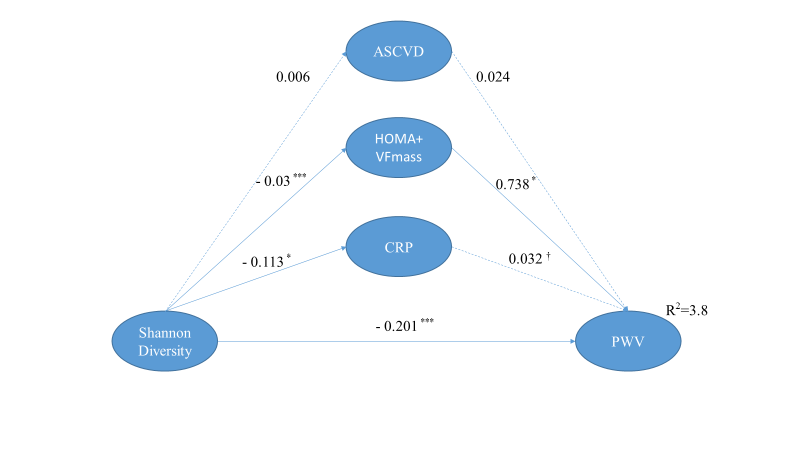
**

**References**

1. Sinclair L, Osman OA, Bertilsson S, Eiler A. Microbial community composition and diversity via 16S rRNA gene amplicons: evaluating the illumina platform. PLoS One 2015;10(2):e0116955.

2. Hair JF, Sarstedt M, Ringle CM, Mena JA. An assessment of the use of partial least squares structural equation modeling in marketing research. Journal of the Academy of Marketing Science 2012;40(3):414-433.

3. Nitzl C, Roldan JL, Cepeda G. Mediation analysis in partial least squares path modeling Helping researchers discuss more sophisticated models. Industrial Management & Data Systems 2016;116(9):1849-1864.

4. Pavlou PA, Fygenson M. Understanding and predicting electronic commerce adoption: An extension of the theory of planned behavior. Mis Quarterly 2006;30(1):115-143.

5. Cohen J. *Statistical power analysis for the behavioral sciences*. Hillsdale, NJ: L. Erlbaum Associates; 1988.
